# Acute femoral shortening for reconstruction of a complex lower extremity crush injury

**DOI:** 10.1007/s11751-018-0311-4

**Published:** 2018-05-23

**Authors:** Philip K. Lim, Bharat Sampathi, Nathan M. Moroski, John A. Scolaro

**Affiliations:** 1 Department of Orthopaedic Surgery, University of California, Irvine, 101 The City Drive South, Orange, CA 92868 USA; 2 School of Medicine, University of California, Irvine, 252 Irvine Hall, Irvine, CA 92697 USA

**Keywords:** Femoral shortening, Knee disarticulation, Traumatic amputation, Open femur, Fracture

## Abstract

Traumatic through-knee or transfemoral amputations with concomitant ipsilateral femoral fractures are extremely rare injuries. The initial goal of management is patient resuscitation and stabilization. Subsequent interventions focus on limb salvage and the creation of a residual limb that can be fitted successfully for a functional lower extremity prosthesis. We present the case of a patient who sustained a traumatic through-knee amputation ipsilateral to an open comminuted femoral fracture. Soft tissue injury prohibited initial primary closure over the distal femoral condyles. A functional residual limb was achieved with acute femoral shortening, maintenance of the femoral condyles and fracture stabilization with a short retrograde intramedullary nail. This approach allowed maintenance of muscular attachments to the femur, soft tissue closure and resulted in a residual limb of acceptable length with a broad weight-bearing surface that was fitted with a prosthesis successfully.

## Introduction

Long bone fractures proximal to traumatic limb amputations are uncommon injuries. They are the result of high-energy trauma or from wartime injuries and are associated with increased complication rates [1]. Treatment is focused on the creation of a functional, well-padded residual limb at the most distal level possible to minimize energy expenditure and maximize function [2]. In this scenario, the decision to proceed with definitive amputation through the fracture site or include fracture fixation can be challenging. The complexity and location of the long bone fracture, as well as degree of soft tissue injury, often dictate the decision.

A through-knee amputation (TKA) is an uncommon amputation level and constitutes less than 2% of all amputations performed in the USA annually [3]. This amputation level has been shown to provide enhanced proprioception, deceased metabolic cost of ambulation, a long lever arm, preservation of the adductor muscle attachments and a broad end weight-bearing surface [4–6].

We present the case of an acute femoral shortening performed through a comminuted open femoral shaft fracture in the setting of an ipsilateral traumatic TKA. The procedure provided the patient with a residual limb of adequate length that possessed many of the advantages of a TKA. The adductor attachments to the residual limb were preserved and a broad end-bearing surface for weight bearing was provided. To the authors’ knowledge, this is the first report of such a technique in the literature.

## Case report

The patient is a 41-year-old male who was involved in a motor vehicle trauma. He was the restrained driver of a large truck that struck another large vehicle. The patient’s left leg was crushed inside the burning cab of the vehicle and traumatically amputated through the knee. There were some contaminated soft tissue and osseous components of the proximal tibia and knee directly within the zone of injury. A circumferential thigh tourniquet was placed in the field by the emergency responders for uncontrolled bleeding from limb.

**Fig. 1:**
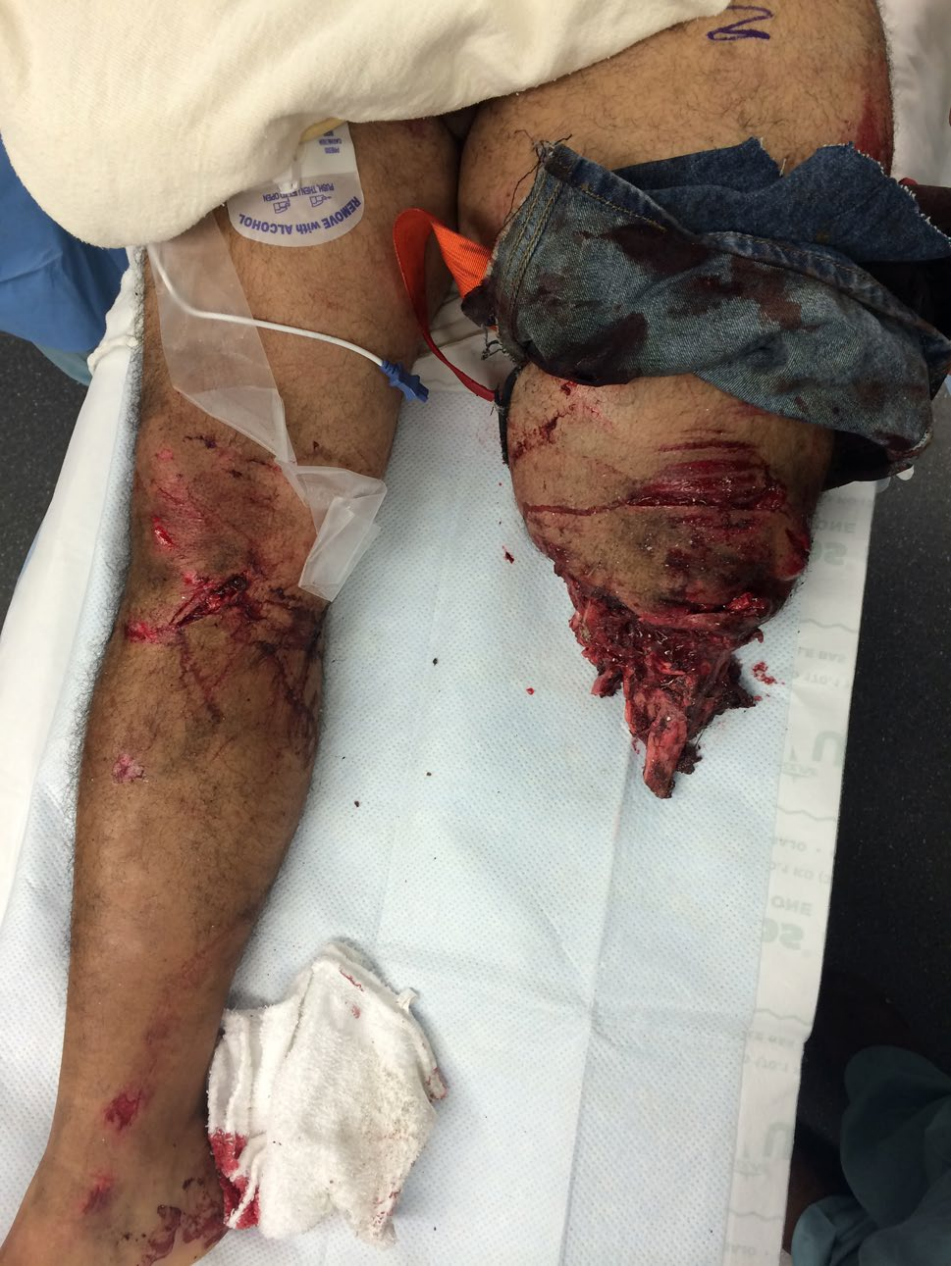
Clinical photograph from trauma resuscitation bay showing appearance of left limb. A circumferential tourniquet is still present on the left limb and the presenting condition of the traumatically amputated limb can be seen

**Fig. 2:**
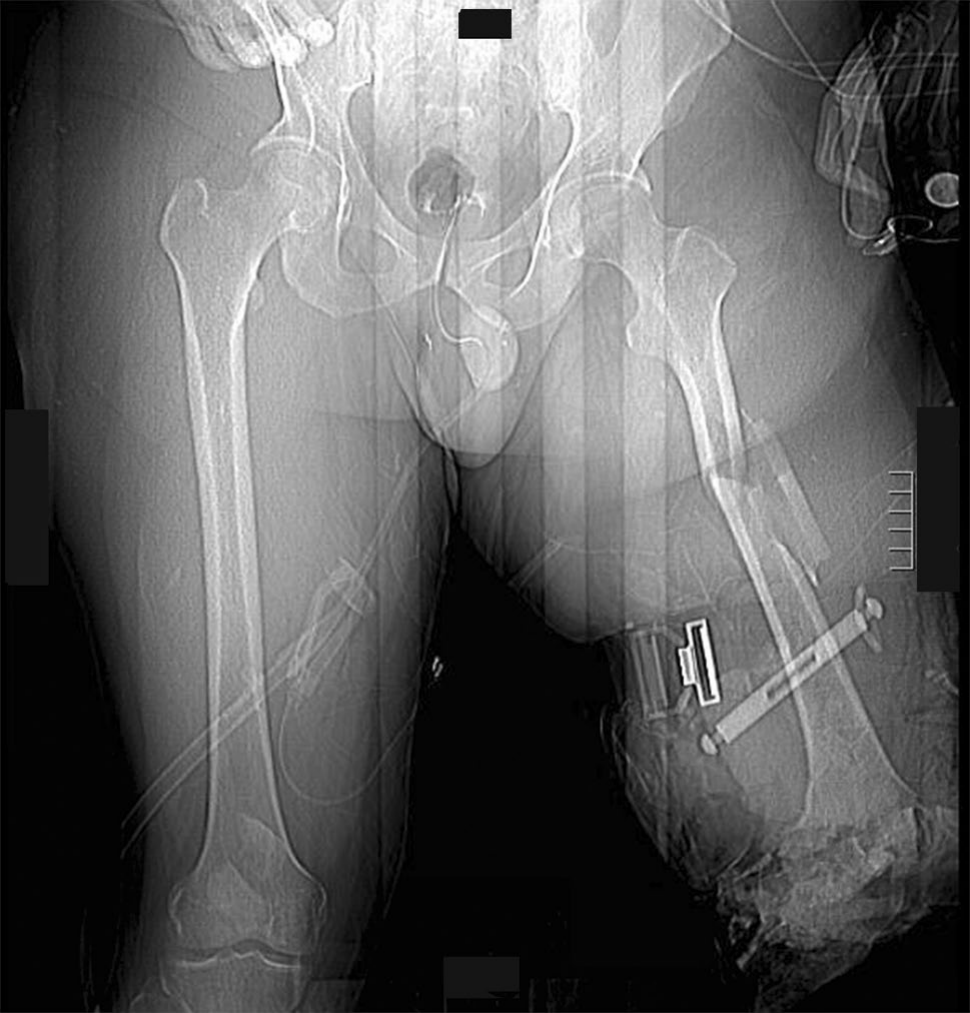
Anterior–posterior scout image from computed tomography study obtained on night of admission showing left comminuted femoral shaft fracture, circumferential tourniquet around leg and traumatic through-knee amputation

In the trauma bay, Advanced Cardiovascular Life Support (ACLS) protocol was followed for initial patient stabilization. Clinical examination revealed a 3-cm open wound along the medial aspect of the mid-thigh just proximal to the applied field tourniquet (Fig. 1). Radiographs taken in the trauma bay demonstrated a comminuted left femoral shaft fracture as well as a near complete amputation of the left lower extremity through the knee (Fig. 2). A closed right patella fracture was the only other injury identified. The patient was brought immediately to the operating room for orthopedic intervention; vascular surgery was consulted and on-call to the operating room.

**Fig. 3:**
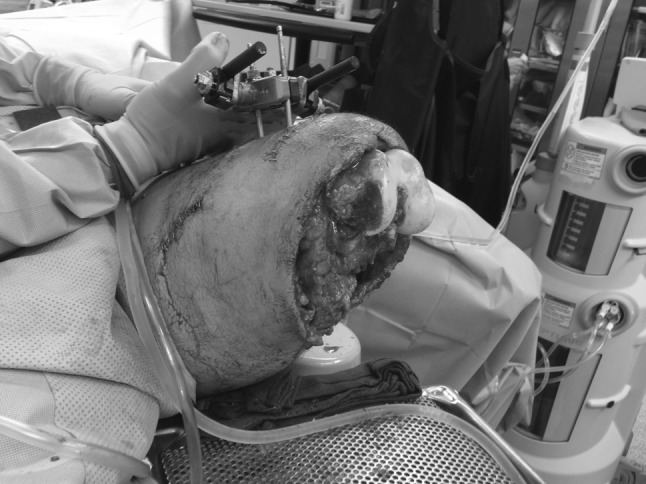
Clinical intra-operative photograph demonstrating level of soft tissue debridement and exposure of distal femoral condyles prior to acute femoral shortening and distal definitive soft tissue closure

In the operating room, the tourniquet was removed and the injury zone explored. The popliteal artery was immediately identified and formally ligated. The remainder of the sciatic nerve was also identified and sharply transected and allowed to retract. Debridement and irrigation of the open femur fracture were performed, and a uniplanar anterior external fixator (Smith and Nephew Inc., Memphis, TN) was applied. The soft tissues about the distal femur were debrided until clean margins were obtained, leaving the distal femoral condyle exposed (Fig. 3). A vacuum-assisted closure (VAC) device (Kinetic Concepts, Inc., San Antonio, TX) was applied over the distal end of the extremity. Forty-eight hours later, the patient returned to the operating room for right patellar fracture fixation and repeat debridement of the left lower extremity traumatic amputation site. A VAC was again placed over the open wound.

On day five, the patient returned to the operating room. The external fixator was removed from the femur. An anterolateral incision was made along the long axis of the femur, and dissection was carried down to the location of the femoral fracture site. The large butterfly fragment was removed and an oscillating saw was used, under saline irrigation, to make flat cuts across the femoral shaft at the distal end of the proximal fragment and the proximal end of the distal fragment. The femur was then acutely shortened approximately 12 cm and held with two clamps through the surgical wound. A standard intercondylar retrograde entry portal was then made for a retrograde nail in the exposed distal femur, and a guidewire was placed across the fracture. The length of the shortened femur was measured, and the canal was sequentially reamed. A Smith and Nephew Trigen 11.5 mm × 250 mm retrograde femoral supracondylar nail was then placed across the fracture (Smith and Nephew Inc., Memphis, TN). The nail was locked distally, and then impacted until direct cortical contact was confirmed at the fracture site. Two proximal interlocking bolts were then placed proximally.

**Fig. 4:**
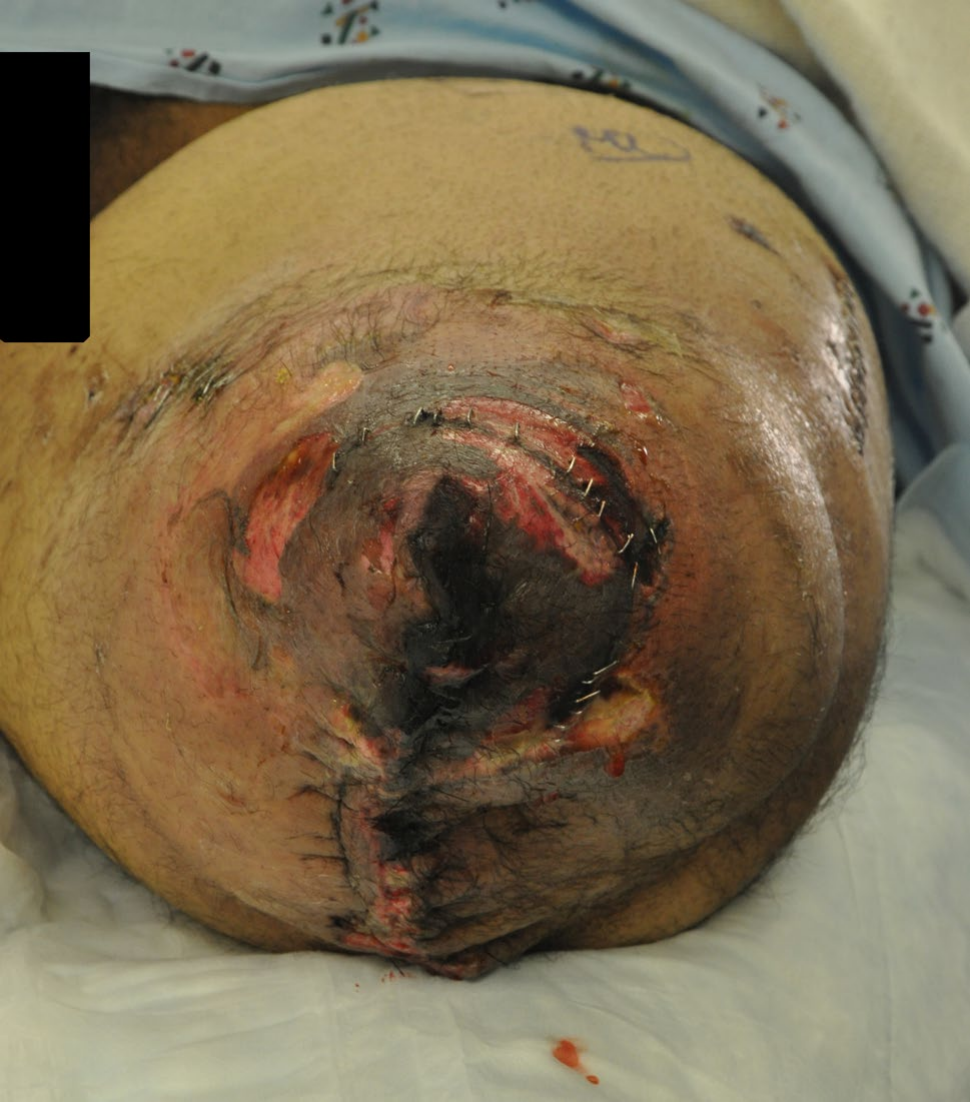
Clinical post-operative photograph demonstrating initial closure of soft tissues about the injured limb after acute femoral shortening. Note the superficial soft tissues which remained compromised due to the crush and thermal injury

**Fig. 5:**
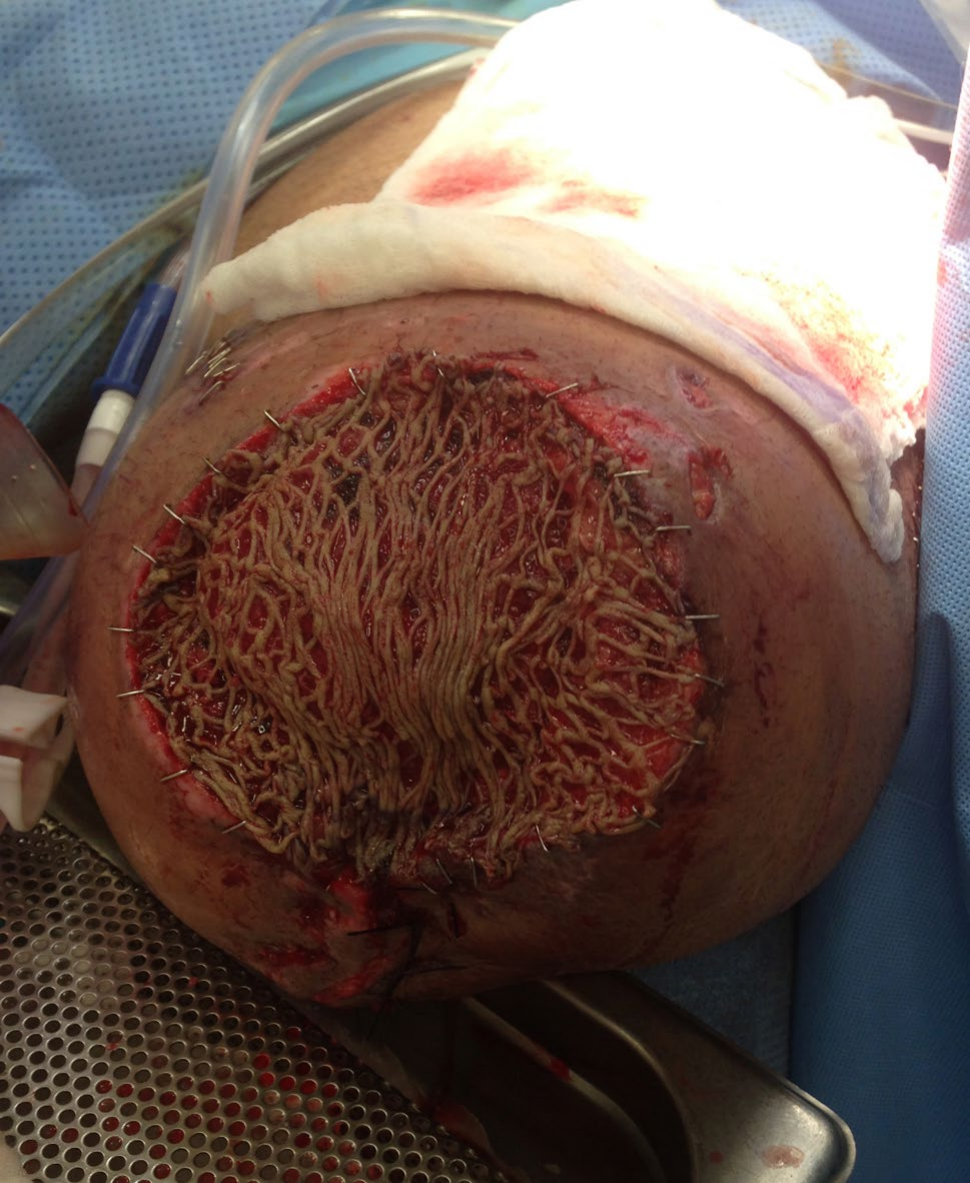
Intra-operative photograph showing complete debridement of nonviable superficial tissue about the residual limb and split-thickness skin grafting over the distal aspect of the limb, placed on the reattached quadriceps and hamstring muscles

At the distal TKA site, the quadriceps tendon was identified; a patellectomy of the remaining fracture fragments was performed and a quadriceps myodesis was performed to the posterior cruciate ligament and medial femoral condyle. Tendons from the semimembranous, semitendinosus and biceps femoris were tenodesed to the quadriceps tendon and soft tissues covering the distal femoral condyles. The adductor attachments to the medial distal femur remained in place. The soft tissues were elevated around the distal femur and closed in layers over the end of the residual limb but resulted in an irregular closure over the distal aspect of the limb with multiple areas of necrotic tissue (from the initial trauma and burn) still remaining (Fig. 4). The patient returned to the operating room 2 days later with the plastic surgery team for superficial debridement of the distal end of the residual limb and split-thickness skin grafting from the ipsilateral thigh. The skin graft was placed over the quadriceps and hamstring muscles that had been pulled over the distal femoral condyles (Fig. 5). The patient remained in the hospital postoperatively and was discharged 1 week later after confirmation that the skin graft had taken without complication.

**Fig. 6:**
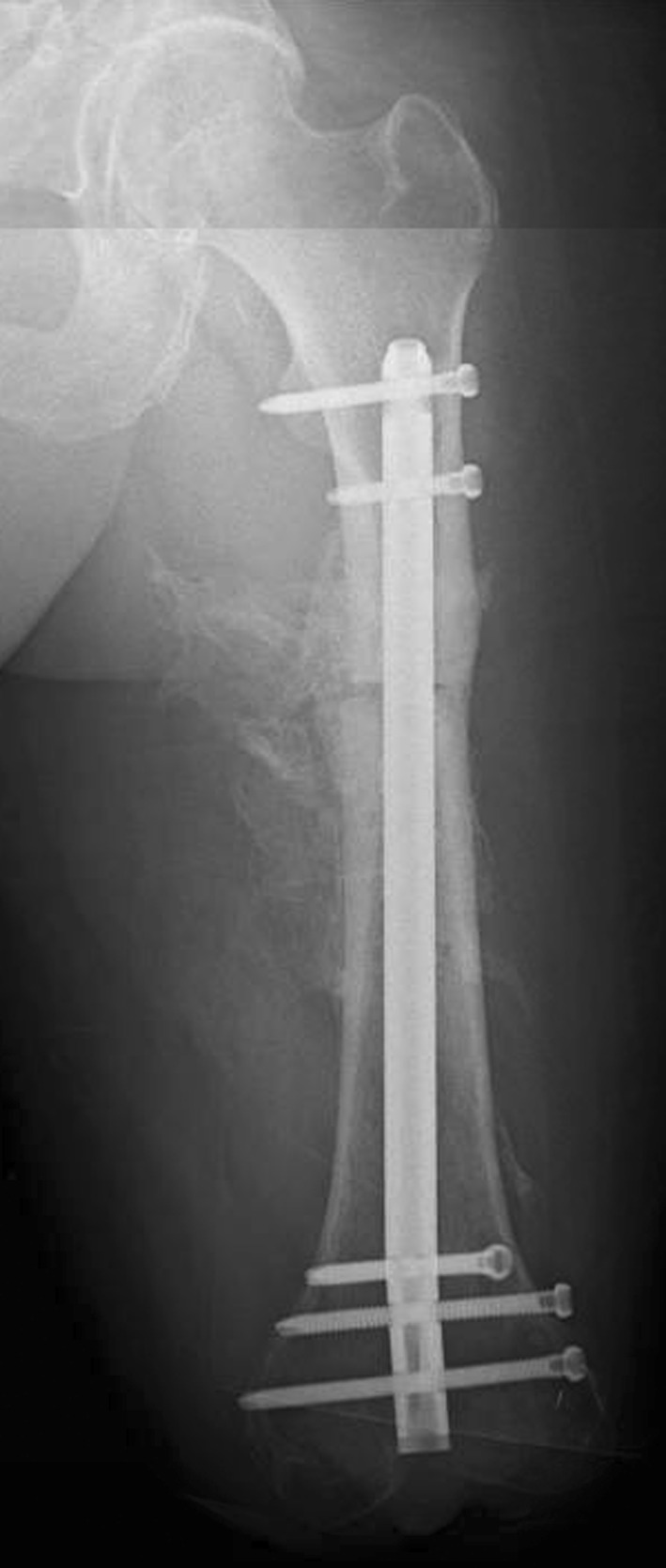
Current anterior-posterior radiograph, 18 months following the acute femoral shortening procedure. Films show stabilization of the residual limb with a short interlocked retrograde femoral intramedullary nail

The patient was followed in the outpatient clinic; all sutures and staples were removed at 4 weeks. After wound healing and maturation had occurred, the patient began prosthesis fitting. He demonstrated excellent control of the limb with no evidence of abduction drift or hip flexion contracture. A modified transfemoral amputation (TFA) prosthesis was successfully fit to the residual limb at 4 months. The patient is currently 18 months out from his injury; his osteotomy has healed with some intramuscular heterotopic ossification (Fig. 6). He reports excellent control of the residual limb and wears his custom prosthesis for the majority of the day. He has occasional phantom limb pain but requires no analgesic medication. He has no areas of soft tissue break down or ulceration along the distal aspect of the residual limb. He ambulates without an assist device and has returned to modified desk work at his original place of employment.

## Discussion

Preservation of residual limb length improves functional outcomes following amputation. In the setting of traumatic limb loss and a more proximal fracture, the fracture location should not define amputation level. Proximal fracture fixation has been shown to preserve joint levels and aid in salvage of residual limb length [1, 7].

Maximization of residual limb length provides many benefits to the patient. Multiple aspects of gait, including walking speed, cadence and stride length have been shown to decrease with more proximal amputations [8]. A recent systematic review and meta-analysis of the available literature have also reported lower quality of life outcome scores as amputation level became more proximal from below knee amputation to TKA and TFA. The authors concluded that limb length should be maximized when amputation is indicated based on the improved quality of life observed [9]. Raichle et al. [10] showed that patients with a more distal amputation level were also more likely to wear a prosthesis as well as use it regularly.

Amputation following trauma constitutes only 16% of all amputations performed annually [2]. Unlike patients who require amputation for chronic illnesses, patients with amputations following trauma are commonly young and active with longer predicted longevity; they constitute almost half of all patients living with a limb amputation [11]. Successful amputation following trauma requires adherence to multiple principles that have been vetted thoroughly during wartime experiences and outlined previously [2].

In the described case, sound amputation principles following trauma were utilized in concert with a novel approach to more proximal fracture management. Regarding limb length, amputation through the proximal aspect of the femoral fracture in this case would have resulted in a residual limb subject to the deforming forces of a subtrochanteric femur fracture: flexion, abduction and external rotation. An extremely short femoral segment would have also left little osseous support for prosthesis fitting. Finally, proximal TFA levels make stable myodesis under physiologic muscle tension increasingly difficult; creation of a robust and stable distal weight-bearing surface can also be challenging at this amputation level.

Essential to the success of this case was the management of the soft tissue envelope about the residual limb. This begins with appropriate handling of the muscles about the amputation, both to balance deforming limb forces and provide adequate distal cushion for the prosthesis. In this case, the majority of the patella had been lost during the traumatic incident so the extensor mechanism was attached to the soft tissue about the distal femur, specifically the remnants of the cruciate ligaments. The posterior muscles were also attached to the distal femur and extensor mechanism to balance and support the limb. Similar to standard amputations, every effort should be made in the traumatic setting to perform the appropriate muscular tenodeses, especially to the quadriceps/extensors and adductors.

The maintenance of the distal articular block (femoral condyles) in this case is similar to what is done in some pediatric amputations to preserve length and prevent bony overgrowth [12]. In some instances, the distal epiphysis is “transplanted” to a more proximal amputated segment to provide a more favorable weight-bearing surface [13]. Unlike many pediatric amputations, commonly required for congenital limb or oncologic conditions, the soft tissues of the adult traumatized limb frequently dictate amputation level. In addition, only in unique circumstances are more distal aspects of an injured limb in the adult able to be moved proximally to achieve length [14].

Acute femoral shortening and stabilization through the open femur fracture provided a novel and successful treatment strategy for this patient. This technique also provided intramedullary bony stabilization, eliminating the presence of a surface implant, and allowed immediate weight bearing on the limb after soft tissue healing and preserved the adductor muscular attachments to the distal femur. Intramedullary femoral stabilization can be performed through an antegrade or retrograde approach depending on the individual injury. In such cases, maintenance of the distal femoral condyles should be considered based on the ability to achieve adequate and appropriate muscular and superficial soft tissue coverage of the limb.

## Conclusion

Traumatic lower extremity amputations with a proximal ipsilateral long bone fracture are rare but severe injuries. The goal of orthopedic management is maximum limb salvage and creation of a functional residual limb for prosthesis fitting. We present the case of a patient with a complex traumatic limb amputation with proximal open femur fracture. The patient was treated with acute femoral shortening through the fracture and retrograde intramedullary fixation. This method of treatment allowed for soft tissue closure, immediate stability and a functional residual limb that was fit easily for a prosthesis.
